# A comprehensive immunohistochemical analysis of IMP2 and IMP3 in 542 cases of ovarian tumors

**DOI:** 10.1186/s13000-023-01300-4

**Published:** 2023-02-06

**Authors:** Kristýna Němejcová, Michaela Kendall Bártů, Romana Michálková, Jana Drozenová, Pavel Fabian, Oluwole Fadare, Jitka Hausnerová, Jan Laco, Radoslav Matěj, Gábor Méhes, Naveena Singh, Simona Stolnicu, Petr Škapa, Marián Švajdler, Ivana Stružinská, David Cibula, Roman Kocian, Sigurd F. Lax, W. Glenn McCluggage, Pavel Dundr

**Affiliations:** 1 Institute of Pathology, First Faculty of Medicine, Charles University and General University Hospital in Prague, Studničkova 2, 12800 Prague 2, Czech Republic; 2 Department of Pathology, 3rd Faculty of Medicine, Charles University, University Hospital Kralovske Vinohrady, 10034 Prague, Czech Republic; 3 Department of Oncological Pathology, Masaryk Memorial Cancer Institute, Brno, Czech Republic; 4 Department of Pathology, University of California San Diego, San Diego, CA USA; 5 Department of Pathology, University Hospital Brno and Medical Faculty, Masaryk University, Brno, Czech Republic; 6 The Fingerland Department of Pathology, Charles University Faculty of Medicine in Hradec Králové and University Hospital Hradec Králové, Hradec Králové, Czech Republic; 7 Department of Pathology and Molecular Medicine, Third Faculty of Medicine, Charles University, Thomayer University Hospital, Prague, Czech Republic; 8 Department of Pathology, Faculty of Medicine, University of Debrecen, Debrecen, 4032 Hungary; 9 Department of Cellular Pathology, Barts Health NHS Trust, and Blizard Institute of Core Pathology, Queen Mary University of London, London, UK; 10 Department of Pathology, University of Medicine, Pharmacy, Sciences and Technology of Targu Mures, Târgu Mureș, Romania; 11 Department of Pathology and Molecular Medicine, Second Faculty of Medicine, Charles University and Motol University Hospital, Prague, Czech Republic; 12 Šikl’s Department of Pathology, The Faculty of Medicine and Faculty Hospital in Pilsen, Charles University, Pilsen, Czech Republic; 13 Gynecologic Oncology Center, Department of Obstetrics and Gynecology, First Faculty of Medicine, Charles University and General University Hospital, 12000 Prague, Czech Republic; 14 Department of Pathology, Hospital Graz II, Graz, Austria, and Johannes Kepler University Linz, Linz, Austria; 15 Department of Pathology, Belfast Health and Social Care Trust, Belfast, UK

**Keywords:** IMP2, IMP3, Ovarian carcinoma, Immunohistochemistry

## Abstract

**Background:**

IMP2 and IMP3 are mRNA binding proteins involved in carcinogenesis. We examined a large cohort of ovarian tumors with the aim to assess the value of IMP2 and IMP3 for differential diagnosis, and to assess their prognostic significance.

**Methods:**

Immunohistochemical analyses with antibodies against IMP2 and IMP3 were performed on 554 primary ovarian tumors including 114 high grade serous carcinomas, 100 low grade serous carcinomas, 124 clear cell carcinomas, 54 endometrioid carcinomas, 34 mucinous carcinomas, 75 mucinous borderline tumors, and 41 serous borderline tumors (micropapillary variant). The associations of overall positivity with clinicopathological characteristics were evaluated using the chi-squared test or Fisher’s Exact test.

**Results:**

We found IMP2 expression (in more than 5% of tumor cells) in nearly all cases of all tumor types, so the prognostic meaning could not be analyzed. The positive IMP3 expression (in more than 5% of tumor cells) was most common in mucinous carcinomas (82%) and mucinous borderline tumors (81%), followed by high grade serous (67%) and clear cell carcinomas (67%). The expression was less frequent in endometrioid carcinomas (39%), low grade serous carcinomas (23%), and micropapillary variant of serous borderline tumors (20%). Prognostic significance of IMP3 could be evaluated only in low grade serous carcinomas in the case of relapse-free survival, where negative cases showed better RFS (*p* = 0.033).

**Conclusion:**

Concerning differential diagnosis our results imply that despite the differences in expression in the different ovarian tumor types, the practical value for diagnostic purposes is limited. Contrary to other solid tumors, we did not find prognostic significance of IMP3 in ovarian cancer, with the exception of RFS in low grade serous carcinomas. However, the high expression of IMP2 and IMP3 could be of predictive value in ovarian carcinomas since IMP proteins are potential therapeutical targets.

**Supplementary Information:**

The online version contains supplementary material available at 10.1186/s13000-023-01300-4.

## Background

The current WHO classification distinguishes between five major histological types of ovarian carcinomas with distinct histological features, immunophenotype, molecular abnormalities, precursors, risk factors, and responsiveness to therapy: high grade serous carcinoma (HGSC), low grade serous carcinoma (LGSC), endometrioid carcinoma (EC), clear cell carcinoma (CCC), and mucinous carcinoma (MC) [1, 2]. Although most ovarian carcinomas are relatively straightforward to diagnose, there may be overlapping histological features between some types, e.g. between HGSC and high-grade EC and ancillary immunohistochemical studies are often employed to aid in the diagnosis. Although diagnostic algorithms for immunohistochemistry based on large studies are available, some tumors may show an overlapping immunohistochemical profile, and additional novel immunohistochemical markers would be helpful [3–6].

The insulin-like growth factor 2 and 3 mRNA binding proteins (IMP2 and IMP3) are closely related members of a family of RNA-binding proteins. They are involved in various biological processes such as development and tumorigenesis, where they also play a role in ovarian carcinomas [7]. In particular, IMPs influence mRNA maturation, localization, translation, and stability via binding to RNA [7]. By these post-transcriptional regulations, IMPs can promote cell proliferation, invasion, and cell migration [7–9]. Overexpression of IMP2 has been described in esophageal, pancreatic, and hepatocellular carcinomas, where it serves as a marker of poor prognosis [10–13]. IMP3 protein is expressed in fetal tissues during embryogenesis, but it is either low or undetectable in adult tissues [9, 14]. Aberrant expression of IMP3 has been described in various malignant tumors, such as pancreatic, colorectal, lung, endocervical, endometrial, renal cell, gastric, urothelial, and breast cancers [8].

We studied a large cohort of ovarian tumors including HGSC, LGSC, CCC, EC, MC, mucinous borderline tumor (MBT) and micropapillary variant of serous borderline tumor (mSBT) with the aim to assess the value of IMP2 and IMP3 for the differential diagnosis. We further investigated the prognostic significance of IMP3 expression in ovarian tumors. IMP2 could not be analyzed because most cases were positive.

## Methods

### Samples

The archives of the pathology departments of the authors were searched for cases diagnosed as primary tubo-ovarian HGSC, LGSC, micropapillary variant of SBT (mSBT; synonymous with the term “noninvasive LGSC” used in the previous WHO classification), MC, MBT, EC, and CCC. In total, 542 cases were selected for immunohistochemical analysis, all of which were reviewed by two gynecological pathologists (PD and KN). The sample set included 114 HGSC, 100 LGSC, 124 CCC, 54 EC, 34 MC, 75 MBT and 41 SBT (only the micropapillary variant), which for the most part represents a dataset of ovarian tumors used in a previous study [15].

### Patient’s clinical characteristics

Clinical data on patient and tumor characteristics at the time of diagnosis and other survival data were obtained retrospectively from medical records. The date of imaging, biopsy confirmation of recurrence or death from the disease was reported as the time of recurrence. If the patient died without determining the cause, the death was recorded as dead of unknown cause (DUC). The clinicopathological and survival characteristics of the 542 patients are summarized in Tables 1 and 2.

**Table 1:** Clinico-pathological characteristics of the 542 ovarian tumors

Characteristics	HGSC (*n* = 114)	LGSC (*n* = 100)	mSBT (*n* = 41)	EC (*n* = 54)	CCC (*n* = 124)	MBT (*N* = 75)	MC (*N* = 34)
**Age (years)**							
Median	60	55	49	54	62	51	58
No. of patients < median	55 (48.2%)	45 (45%)	15 (35.7%)	25 (46.3%)	57 (46.0%)	35 (46.7%)	16 (47.1%)
No. of patients ≥ median	59 (51.8%)	47 (47%)	18 (42.8%)	25 (46.3%)	65 (52.4%)	35 (46.7%)	16 (47.1%)
NA	0 (0%)	8 (8%)	9 (21.5%)	4 (7.4%)	2 (1.6%)	5 (6.6%)	2 (5.8%)
**FIGO**							
IA	1 (0.9%)	2 (2%)	3 (7.3%)	20 (37.0%)	40 (32.3%)	59 (78.7%)	14 (41.2%)
IB	4 (3.5%)	1 (1%)	3 (7.3%)	6 (11.1%)	1 (0.8%)	0 (0%)	1 (2.9%)
IC	4 (3.5%)	11 (11%)	5 (12.2%)	19 (35.2%)	39 (31.5%)	14 (18.6%)	11 (32.4%)
II (IIA + IIB)	7 (6.1%)	6 (6%)	2 (4.9%)	3 (5.6%)	10 (8%)	0 (0%)	1 (2.9%)
III	68 (59.6%)	52 (52%)	16 (39%)	3 (5.6%)	24 (19.4%)	0 (0%)	6 (17.6%)
IV	28 (24.6%)	2 (2%)	0 (0%)	1 (1.9%)	1 (0.8%)	1 (1.3%)	1 (2.9%)
NA	2 (1.8%)	26 (26%)	12 (29.3%)	2 (3.7%)	9 (7.3%)	1 (1.3%)	0 (0%)
**T stage**							
T1a	3 (2.6%)	2 (2%)	3 (7.3%)	21 (38.9%)	40 (32.3%)	59 (78.7%)	14 (41.2%)
T1b	5 (4.4%)	1 (1%)	3 (7.3%)	6 (11.1%)	1 (0.8%)	0 (0%)	1 (2.9%)
T1c	5 (4.4%)	11 (11%)	5 (12.2%)	19 (35.2%)	41 (33%)	14 (18.6%)	11 (32.4%)
T2	13 (11.4%)	6 (6%)	2 (4.9%)	3 (5.6%)	12 (9.7%)	0 (0%)	1 (2.9%)
T3	85 (74.6%)	53 (53%)	16 (39%)	3 (5.6%)	22 (17.7%)	1 (1.3%)	7 (20.6%)
NA	3 (2.6%)	27 (27%)	12 (29.3%)	2 (3.7%)	8 (6.5%)	1 (1.3%)	0 (0%)
**Lymph node involvement**							
N0	29 (25.4%)	17 (17%)	12 (29.3%)	40 (74.1%)	53 (42.7%)	9 (12%)	12 (35.3%)
N1	44 (38.6%)	20 (20%)	3 (7.3%)	1 (1.9%)	7 (5.7%)	0 (0%)	3 (8.8%)
NA	41 (36%)	63 (63%)	26 (64.4%)	13 (24.0%)	64 (51.6%)	66 (88%)	19 (55.9%)

*HGSC* high grade serous carcinoma, *LGSC* low grade serous carcinoma, *mSBT* micropapillary serous borderline tumor, *EC* endometrioid carcinoma, *CCC* clear-cell carcinoma, *MBT* mucinous borderline tumor, *MC* mucinous carcinoma, *NA* data not available

**Table 2:** Overview of the survival characteristics of the study cohort (cases with available follow-up)

	HGSC (*n* = 110)	LGSC (*n* = 81)	mSBT (*n* = 27)	EC (*n* = 50)	CCC (*n* = 106)	MBT (*N* = 63)	MC (*N* = 27)
**Follow-up (duration in months)**							
Median	38	43	58	64	37	46	10
Mean	43	55	56	65	46	50	34
Max	251	320	189	171	198	162	123
**Survival status**							
NED	26 (23.6%)	37 (45.7%)	17 (63.0%)	47 (94%)	61 (57.5%)	61 (96.8%)	17 (62.9%)
AWD	60 (54.5%)	24 (29.6%)	6 (22.2%)	2 (4%)	19 (17.9%)	0 (0%)	7 (25.9%)
DOD	24 (21.8%)	11 (13.6%)	3 (11.1%)	1 (2%)	13 (12.3%)	0 (0%)	2 (7.5%)
DTC	0 (0%)	1 (1.2%)	0 (0%)	0 (0%)	3 (2.8%)	0 (0%)	0 (0%)
DUC	0 (0%)	7 (8.6%)	1 (3.7%)	0 (0%)	7 (6.6%)	2 (3.2%)	1 (3.7%)
DOC	0 (0%)	1 (1.2%)	0 (0%)	0 (0%)	3 (2.8%)	0 (0%)	0 (0%)

*HGSC* high grade serous carcinoma, *LGSC* low grade serous carcinoma, *mSBT* micropapillary serous borderline tumor, *EC* endometrioid carcinoma, *CCC* clear-cell carcinoma, *MBT* mucinous borderline tumor, *MC* mucinous carcinoma, *NED* no evidence of disease, *AWD* alive with disease, *DOD* death of diagnosis, *DTC* death of treatment complications, *DUC* death of unknown cause, *DOC* death of other cause

### Immunohistochemical analysis

The immunohistochemical analysis was performed using tissue microarrays (TMAs) as described in our previous study [15]. The TMA technique was used based on previous studies which described IMP2 and IMP3 expression in ovarian tumors, in line with the recommendation of a review study designed by Burdeleski et al., which recommended the TMA as a convenient technique for evaluating IMP3 expression [8]. The expression of the following antibodies was analyzed in each case: IMP2, using two clones since the experience with these antibodies is rather limited (clone OTI3F9, dilution 1:200, Novus Biologicals, USA; clone EPR6741(B), dilution 1:200, Abcam, USA), and IMP3 (clone 69.1, dilution 1:200, Glostrup Denmark), with antigen retrieval HIER, and detection by EnVision FLEX, Dako. For both IMP2 clones the staining was performed by the Ventana BenchMark ULTRA instrument (Roche, Basel, Switzerland) with the OptiView detection kit. Normal ovarian surface epithelium served as negative control, while lymph follicles in lymph nodes were used as a positive control.

The expression of all markers was double-blindly evaluated by at least two of three pathologists in various combinations (KN, PD, MKB).

Cytoplasmic expression for all markers was assessed semiquantitatively (the overall percentage of positive cells). Cases were classified based on the overall percentage of positive cells as negative (entirely negative or arbitrarily set as ≤5% of positive tumor cells) or positive (more than 5% positive tumor cells).

### Statistical analysis

The software R (version 4.0.2, https://www.r-project.org/) and Statistica (TIBCO Statistica 13.5.0, CA, USA) were used to perform the statistical analyses.

Associations of overall positivity (≤ 5% of positive tumor cells = negative, versus 6–100% of positive tumor cells = positive) with clinicopathological characteristics were evaluated using the chi-squared test or Fisher’s Exact test.

Overall survival (OS), relapse-free survival (RFS), local recurrence-free survival (LFS), and metastasis-free survival (MFS) analyses were performed. If the patient did not show any of the monitored events, the case was censored in the analysis to the date of the last follow-up. Survival analyses were plotted using Kaplan-Meier model and log-rank statistics were used to test for differences between positive and negative cases.

All tests were two-sided and a *p*-value of less than 0.05 was considered as significant.

## Results

The clinicopathological and survival characteristics of the 542 patients are summarized in Tables 1 and 2. The immunohistochemical results in relation to the individual tumor types are summarized in Table 3. Overall, IMP2 (both clones) showed a higher percentage of positivity than IMP3 in all tumor types (Table 3). We also tried to use other different cut-offs (5, 10, and 50%) for differential diagnostic purposes between ovarian tumors, and the results are stated in Table 4.

**Table 3:** Expression of IMP3 and IMP2 [clone OTI3F9 and clone EPR6741(B)] in the different tumor types

Marker	HGSC (*N* = 114)	LGSC (*N* = 100)	mSBT (*N* = 41)	EC (*N* = 54)	CCC (*N* = 124)	MBT (*N* = 75)^a^	MC (*N* = 34)^a^
**IMP3**							
Negative (1–5%)	38 (33%)	77 (77%)	33 (80%)	33 (61%)	41 (33%)	14 (19%)	6 (18%)
Any positivity (> 5%)	76 (67%)	23 (23%)	8 (20%)	21 (39%)	83 (67%)	61 (81%)	28 (82%)
Positivity (> 5–10%)	5 (4%)	11 (11%)	3 (7.5%)	2 (4%)	4 (3%)	2 (3%)	1 (3%)
Positivity (> 10–50%)	23 (20%)	8 (8%)	2 (5%)	3 (5%)	27 (22%)	15 (20%)	6 (17%)
Positivity (> 50–100%)	48 (42%)	4 (4%)	3 (7.5%)	16 (30%)	52 (42%)	44 (58%)	21 (62%)
**IMP2 (clone OTI3F9)**							
Negative (1–5%)	2 (2%)	2 (2%)	0 (0%)	5 (9%)	15 (12%)	3 (4%)	1 (3%)
Any positivity (> 5%)	112 (98%)	98 (98%)	41 (100%)	49 (91%)	109 (88%)	70 (96%)	31 (97%)
Positivity (> 5–10%)	1 (1%)	2 (2%)	0 (0%)	1 (2%)	3 (2%)	0 (0%)	1 (3%)
Positivity (> 10–50%)	4 (3%)	8 (8%)	0 (0%)	7 (13%)	12 (10%)	1 (1%)	4 (13%)
Positivity (> 50–100%)	107 (94%)	88 (88%)	41 (100%)	41 (76%)	94 (76%)	69 (95%)	26 (81%)
**IMP2_clone EPR6741(B)**							
Negative (1–5%)	0 (0%)	2 (2%)	0 (0%)	13 (24%)	11 (9%)	3 (4%)	3 (9%)
Any positivity (> 5%)	114 (100%)	98 (98%)	41 (100%)	41 (76%)	113 (91%)	70 (96%)	29 (91%)
Positivity (> 5–10%)	1 (1%)	2 (2%)	0 (0%)	0 (0%)	2 (2%)	0 (0%)	1 (3%)
Positivity (> 10–50%)	9 (8%)	7 (7%)	0 (0%)	10 (19%)	4 (3%)	6 (8%)	3 (9%)
Positivity (> 50–100%)	104 (91%)	89 (89%)	41 (41%)	31 (57%)	107 (86%)	64 (88%)	25 (78%)

*HGSC* high grade serous carcinoma, *LGSC* low grade serous carcinoma, *mSBT* micropapillary serous borderline tumor, *EC* endometrioid carcinoma, *CCC* clear-cell carcinoma, *MBT* mucinous borderline tumor, *MC* mucinous carcinoma

^a^expression of both IMP2 clones is not available for 2 MBT and 2 MC cases

**Table 4:** The difference in the number of cases in the two categories in relation to the cut-off of IMP3 overall positivity. *P*-values are based on chi-squared test

	cut-off = 5%			cut-off = 10%			cut-off = 50%		
	N positive	N negative	*p*-value	N positive	N negative	*p*-value	N positive	N negative	*p*-value
**HGSC x LGSC**			**< 0.001**			**< 0.001**			**< 0.001**
HGSC	76 (67%)	38 (33%)		71 (62%)	43 (38%)		48 (42%)	66 (58%)	
LGSC	23 (23%)	77 (77%)		12 (12%)	88 (88%)		4 (4%)	96 (96%)	
**HGSC x CCC**			0.965			0.819			0.979
HGSC	76 (67%)	38 (33%)		71 (62%)	43 (38%)		48 (42%)	66 (58%)	
CCC	83 (67%)	41 (33%)		79 (64%)	45 (36%)		52 (42%)	72 (58%)	
**HGSC x EC**			**< 0.001**			**0.001**			0.119
HGSC	76 (67%)	38 (33%)		71 (62%)	43 (38%)		48 (42%)	66 (58%)	
EC	21 (39%)	33 (61%)		19 (35%)	35 (65%)		16 (30%)	38 (70%)	
**EC x CCC**			**< 0.001**			**< 0.001**			0.120
EC	21 (39%)	33 (61%)		19 (35%)	35 (65%)		16 (30%)	38 (70%)	
CCC	83 (67%)	41 (33%)		79 (64%)	45 (36%)		52 (42%)	72 (58%)	
**MC x HGSC**			0.079			0.064			**0.044**
MC	28 (82%)	6 (18%)		27 (79%)	7 (21%)		21 (62%)	13 (38%)	
HGSC	76 (67%)	38 (33%)		71 (62%)	43 (38%)		48 (42%)	66 (58%)	
**MC x EC**			**< 0.001**			**< 0.001**			**0.003**
MC	28 (82%)	6 (18%)		27 (79%)	7 (21%)		21 (62%)	13 (38%)	
EC	21 (39%)	33 (61%)		19 (35%)	35 (65%)		16 (30%)	38 (70%)	
**MC x CCC**			0.082			0.084			**0.040**
MC	28 (82%)	6 (18%)		27 (79%)	7 (21%)		21 (62%)	13 (38%)	
CCC	83 (67%)	41 (33%)		79 (64%)	45 (36%)		52 (42%)	72 (58%)	

*HGSC* high grade serous carcinoma, *LGSC* low grade serous carcinoma, *mSBT* micropapillary serous borderline tumor, *EC* endometrioid carcinoma, *CCC* clear-cell carcinoma, *MBT* mucinous borderline tumor, *MC* mucinous carcinoma

### IMP2

In all tumor types there was a strong positive correlation between the two clones (HGSC: R = 0.582, *p* <  0.001; LGSC: R = 0.867, *p* <  0.001; SBT: R = 0.828, *p* = 0.004; EC: R = 0.803, *p* <  0.001; CCC: R = 0.775, *p* <  0.001; MC: R = 0.507, *p* <  0.001, MBT: R = 0.658, *p* <  0.001).

All tumor types showed higher expression of IMP2 (for both antibodies) compared to IMP3 (Table 3).

### IMP3

**Fig. 1:**
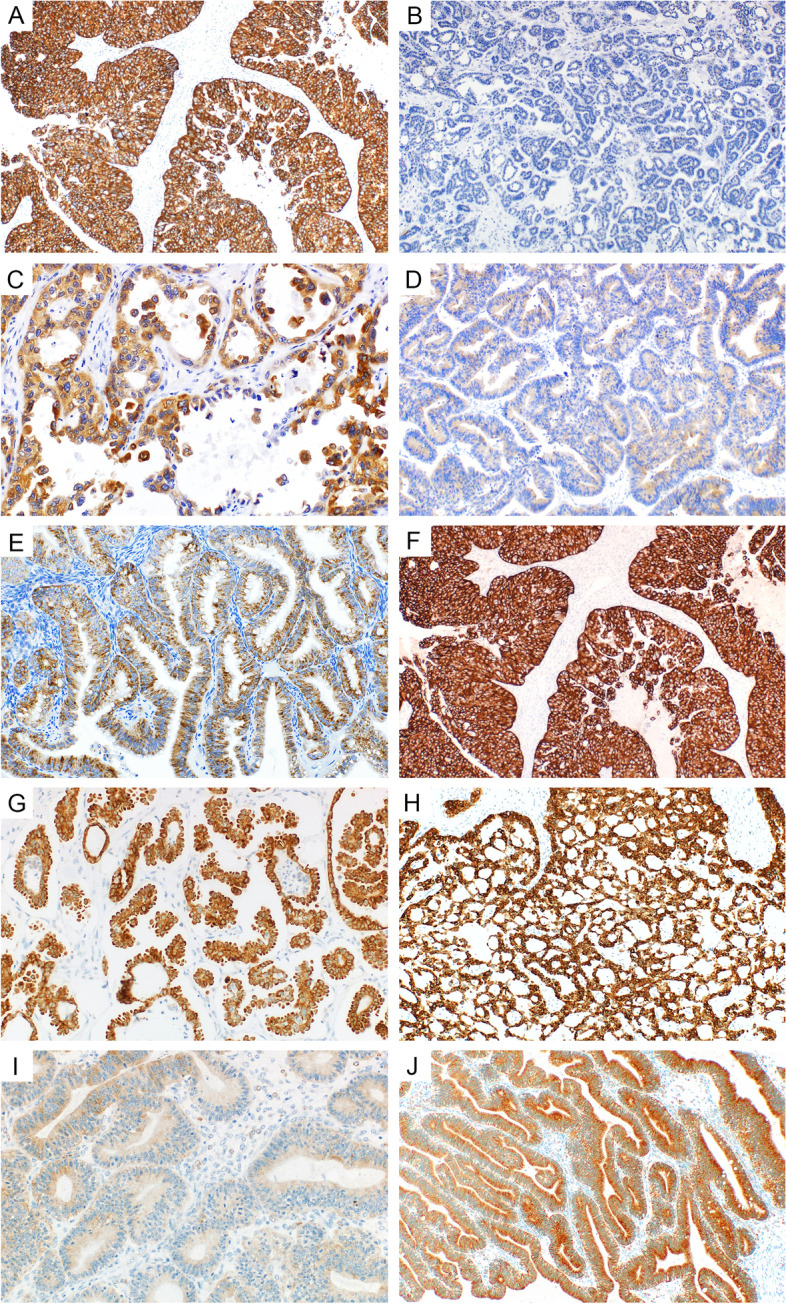
**A** – **E** Expression of IMP3: **A** - in HGSC (100x), **B** – in LGSC (100x), **C** – in CCC (200x), **D** – in EC (100x). **F**-**G** Expression of IMP2 (IGF2): **F** - in HGSC (100x), **G** – in LGSC (200x), **H** – in CCC (100x), **I** - in EC (200x), **J** – in MC (100x)

IMP3 expression in the various tumor types using 3 different cut-offs (5, 10, and 50%) is detailed in Table 3. The highest positivity of IMP3 according to the percentage of positive cells was found in MC (82% positive cases, 62% cases with expression in > 50% of tumor cells) and MBT (81% positive cases, 58% cases with expression in > 50% of tumor cells). IMP3 immunoreactivity was significantly higher in MC with expansile invasion compared to MC with infiltrative invasion (*p* = 0.015). High expression was also found in HGSC (67% positive cases, 42% cases with expression in > 50% of tumor cells), and CCC (67% positive cases, 42% cases with expression in > 50% of tumor cells). The lowest positivity was found in LGSC and mSBT with similar results (LGSC: 23% positive cases, 4% cases with expression in > 50% of tumor cells; mSBT: 20% positive cases, 7.5% cases with expression in > 50% of tumor cells). The EC group showed intermediate levels of IMP3 expression compared to the other tumor types (39% positive cases, 30% cases with expression in > 50% of tumor cells) (Fig. 1).

### Prognostic analysis

Survival analysis was performed only for the tumor types with sufficient number of events of interest (see Supplementary Table S1 for a detailed overview of the sample size and results of time-to-event analyses in the individual subsets).

**Fig. 2:**
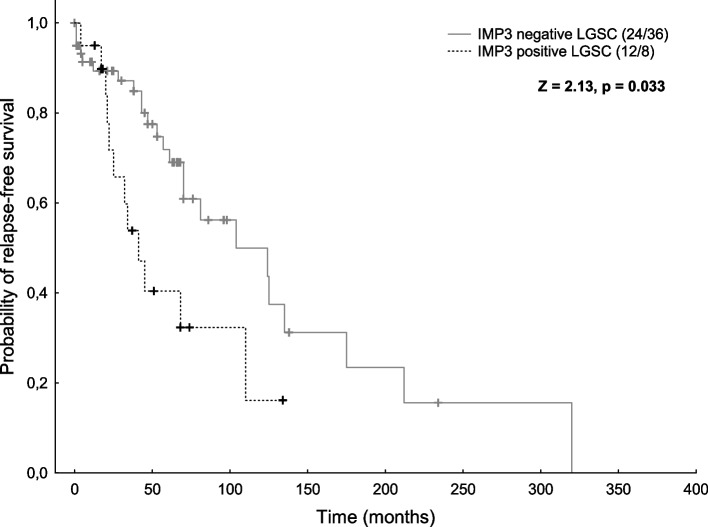
Kaplan-Meier curves for RFS of LGSC patients in relation to expression of  IMP3

The only significant difference was detected in LGSC in case of relapse-free survival, where negative cases showed better RFS (*p* = 0.033, Fig. 2). None of the other clinicopathological correlations between the selected parameters and IMP2/IMP3 expression showed statistically significant results. In HGSC there was a trend towards a positive correlation between T stage and IMP3 expression, but it did not reach statistical significance (*p* = 0.054, Supplementary Table S2, note the limited sample size of the negative cases for both IMP2 clones).

## Discussion

In the female genital tract, the expression of IMPs has, so far, been mostly investigated in ovarian and endometrial tumors. In endometrial lesions IMP3 expression has been found in benign endometrium, atypical endometrial hyperplasia, and endometrial carcinomas, with the highest expression in serous carcinomas [16–21]. Li et al. found IMP3 positivity in more than 50% of tumor cells in 93% of 42 serous carcinomas, and in only 7% of 8 endometrioid carcinomas and concluded that IMP3 may serve as a helpful biomarker in the distinction between these two tumor types (*p* <  0.001) [22]. Zheng et al. found IMP3 positivity in more than 50% of tumor cells in 94% of serous carcinomas and 49% of clear cell carcinomas compared to 3% of endometrioid carcinomas and complete negativity in 8 mucinous endometrial carcinomas [19]. They concluded that IMP3 is a highly sensitive marker for both endometrial serous and clear cell carcinomas. Mhawech-Fauceglia et al. found IMP3 positivity (in more than 5% of tumor cells) in 100 and 12% of serous and endometrioid endometrial carcinomas, respectively and suggested the combination of IMP3 and PTEN as a useful panel for distinguishing between these two tumor types (*P* <  0.001) [16]. In a subsequent study on 401 cases, the same authors found 78% of serous carcinomas, 57% of clear cell carcinomas and 15% of endometrioid carcinomas IMP3 positive by using a combined scoring system for intensity and percentage of expression [17]. Zaidi et al. found IMP3 positivity in 64% of endometrial serous carcinomas and 19% of grade 3 endometrioid carcinomas using 5% cut-off for positivity, and suggested a panel of p53, p16, IMP3, ER and PR as best for distinguishing serous from endometrioid carcinoma [18].

Concerning IMP3 expression in ovarian tumors, the published literature provides less clear evidence. Studies which have focused on IMP3 expression in HGSC have shown variable results. In 6 studies with a total of 526 cases of HGSC IMP3 positivity ranged from 23 to 68% (average 49%) [14, 23–27]. Three other studies found IMP3 expression in 45 to 93% of 110 “serous carcinoma cases”, but the type of serous carcinoma was not further specified [8, 28, 29]. Our data showed IMP3 expression in 67% of HGSC, which is consistent with studies that used the same cut-off for positivity and the same antibody clone [14, 28]; there is only one study which found a lower expression [25]. The explanation for such a wide range of IMP3 expression in the various studies is not clear but could be related to the different cut-offs for positivity used. Two studies used 10% of positive tumor cells as a cut off [8, 26], while the remaining studies used different scoring systems, mostly taking into account staining intensity as well as percentage of positive tumor cells [24, 27, 29, 30].

Unlike HGSC, expression of IMP3 in LGSC has not yet been studied in detail. One study analyzed the RNA expression levels in ovarian tumors including three cases of LGSC, but without correlation with immunohistochemical expression [31]. In our study, IMP3 expression was present in 23% of LGSC and 20% mSBT. We identified four studies analyzing IMP3 expression in SBT, with 125 cases in total, but only one of them mentions the mSBT subtype. In three of these studies all SBT cases were IMP3 negative [23, 24, 27, 28]. One study on 84 SBT, including 32 cases with a micropapillary pattern, reported absent or weak IMP3 expression in 34%, medium IMP3 expression in 57%, and strong IMP3 expression in 9% of cases [23]. Compared to previous studies, our study included only mSBT and, considering our results, expression of IMP3 in mSBT seems to differ from typical SBT.

IMP3 expression in ovarian EC has been investigated in 4 studies (comprising a total of 265 cases), but the data is equivocal with a wide range of positivity varying between 2 and 40% [8, 25–27]. However, the studies used different cut-offs for positivity; one study used a 5% cut-off (27% of positive cases), two a 10% cut-off (2 and 33% of positive cases), and the other combined scoring systems (40% of positive cases). In our study, IMP3 expression was found in 39% of cases of EC.

Four studies with a total of 337 cases investigated the expression of IMP3 in CCC and the expression ranged from 52 to 86%, which is in accordance with our result of 67% [25–27, 32]. These studies also used different cut-offs for positivity; one study used a 5% cut-off (52% of positive cases), two a 10% cut-off (63 and 86% of positive cases, respectively), and one study a combined scoring system (75% of positive cases).

We identified only three studies focusing on IMP3 expression in ovarian mucinous tumors, two of which included MC only [23, 25, 33]. In one study, the authors found IMP3 expression in 86% of 30 MC using a cut-off of 5% [25]. Another study on 250 ovarian mucinous tumors found any extent of IMP3 expression in 12% of benign tumors, 91% of MBT, and 100% of MC [33]. In another study on 140 MBT, moderate or strong expression was found in 66% of cases when using a combined immunoreactive score [23]. The results of our study showed a similar frequency of IMP3 expression in MBT and MC. IMP3 expression of any extent was found in 81% MBT and 82% MC, which is consistent with the above-mentioned results.

So far, only one study has examined the value of IMP3 expression in the differential diagnosis of ovarian carcinomas, and the authors suggested IMP3 to be an additional diagnostic marker for ovarian CCC [26]. The authors investigated 336 ovarian carcinomas (including 132 CCC, 103 HGSC, and 116 EC) and found IMP3 positivity in 86% of CCC, 23% of HGSC, and 2% of EC cases. IMP3 expression was significantly higher in CCC compared to HGSC and EC (*p* <  0.01) and, therefore, it was concluded that IMP3 is a useful marker to distinguish CCC from HGSC and EC. The study used a different antibody clone and a different cut-off for positivity (more than 10% of positive tumor cells) compared to ours, which did not find significant differences between CCC and HGSCs [26]. In our study, using the 5% cut-off, we did not find significant differences between CCC and HGSC. When using 3 different cut-offs (5, 10, and 50%), we found differences between CCC and EC for the cut-off 10% which is in concordance with the study just mentioned but there was no difference between HGSC and CCC (Table 4).

To date, only three studies have focused on the immunohistochemical expression of IMP2 in tumors of the female genital tract. Two of those studied endometrial tumors, while the other focused on ovarian tumors. Both endometrial studies suggested IMP2 as a marker which can be used in the differential diagnosis between endometrioid and serous carcinomas [34, 35]. One of these studies suggested the use of IMP2 in a diagnostic panel which would allow for the discrimination between endometrioid (G1 and G2) and serous carcinoma based on an H-score (IMP2 ≥ 115) [34]. However, using an H-score as a discriminator is not very feasible in diagnostic practice, and the sensitivity for differentiating grade 3 endometrioid carcinoma from serous carcinoma was lower. The other study investigated IMP2 expression in 227 endometrial carcinomas, and 93 cases of benign endometrium [35]. The authors found diffuse expression (in > 95% tumor cells) in all 27 cases of serous carcinomas, but all endometrioid carcinomas showed IMP2 expression in less than 75% of tumor cells (in 32% of tumors the expression was in < 5% of tumor cells). They concluded that IMP2 expression in < 75% of the tumor cells can help distinguish endometrioid from serous carcinomas.

Only one study examined the immunoreactivity of IMP2 in epithelial ovarian tumors and included ovarian transitional cell carcinoma, which is now considered a pattern of HGSC [36]. By using a combined score, they found a lower percentage of positive cells in Brenner tumors compared to transitional cell carcinomas. Two other studies examined protein expression profiling and found IMP2 expression in 89% (39/44) of HGSC and in 11% (2/18) of EC [37, 38].

The results of our study showed high IMP2 expression (of both clones) in all tumor types, which was higher compared to IMP3 expression. The highest IMP2 expression was detected in mSBT (showing 100% for both clones), and HGSC (both clones in the range of 98–100%). The expression was slightly lower, but still high, in EC (76–91%), LGSC (98%), and CCC (88–91%). In mucinous tumors the expression was 96% in MBT and 91–97% in MC. We did not find any statistically significant differences between the expression of IMP2 and IMP3 in MBT and MC, or in SBT and LGSC.

Although the expression of IMP3 showed statistically significant differences between some tumor types in our study, the value for differential diagnosis of ovarian tumors seems to be rather limited. In particular, the assessment of cut-offs for positivity seems to be problematic for most tumor types in diagnostic practice. The only exception seems to be the differences in expression between HGSC and LGSC, especially when considering a 50% cut-off, which was present in 42% of HGSC but only in 4% of LGSC. However, the practical value of this finding seems to be limited as the morphological distinction between HGSC and LGSC is usually straightforward and there are other useful antibodies such as p53, p16, and Ki67. Furthermore, IMP3 is not useful in the differential diagnosis between HGSC and grade 3 EC, which can also be problematic in some cases. Our study showed no practical use for IMP2 in the differential diagnosis of epithelial ovarian tumors.

Most studies of IMP3 and IMP2 expression in ovarian tumors have focused on its prognostic importance, but the results are not conclusive. The poor prognostic impact of IMP3 expression has been confirmed only in ovarian CCC [25, 32]. Our study showed no correlation between IMP3 expression and various clinicopathological parameters or patient outcomes (with respect to OS, RFS, LFS, and MFS), except for LGSC, which showed a significant difference in the case of RFS, seeing as negative cases had better RFS (*p* = 0.033). In mucinous tumors, high IMP2/EPR6741(B) expression was associated with favorable LFS, but the low number of events in our MC sample set limited the survival analyses. However, the high expression of IMP2 and IMP3 in ovarian carcinomas may gain further interest since IMP proteins are potential therapeutical targets [7]. One study focused on colorectal cancer reported that IMP3 directly regulates MEKK1 mRNA, and thus activates the MEK/ERK pathway [39]. The authors suggest that inhibiting IMP3, possibly in combination with a MEK1 inhibitor, may provide new potential therapeutic strategies for colorectal cancer treatment. Another study demonstrated that inhibition of IMP3 expression in cell lines of neuroendocrine tumors leads to downregulation of *EGFR* and Ki-67, proteins associated with cell proliferation, and these authors suggest IMP3 as a promising therapeutic target [40]. So far only one study described the inhibition of IMP2 in vitro and in vivo (in colorectal carcinoma and hepatocellular cell lines, zebrafish embryos) [7]. The results showed reduced growth of xenotransplanted tumor cells, which supports the theory that IMP2 represents a druggable target.

## Conclusion

The results of our study suggest only a limited value of IMP2 and IMP3 for the differential diagnosis of ovarian epithelial tumors. Although we found differences in IMP3 expression between HGSC and LGSC, the practical value of this for differential diagnosis seems to be rather limited. We did not confirm the prognostic significance of IMP3 expression in ovarian epithelial tumors, with the exception of LGSC, where negative cases were associated with longer RFS. This is in contrast to other solid tumors. IMP2 could not be analyzed with respect to its prognostic impact due to the high levels of positivity in most cases. Nevertheless, IMP3 and IMP2 expression could be of predictive value, should inhibitors of the RNA-binding protein become therapeutic.

## Supplementary Information

**Additional file 1: Table S1.** Overview of the sample size and results of survival analyses for each tumor type. **Table S2.** Correlations between the studied markers and the selected clinico-pathological parameters for different tumor types.

## Data Availability

All data generated or analyzed during this study is included in this published article (and its Supplementary information files).
