# Supplementary material for: A comprehensive immunohistochemical analysis of IMP2 and IMP3 in 542 cases of ovarian tumors
Source: Diagn Pathol. 2023 Feb 6;18:15. doi: 10.1186/s13000-023-01300-4 (PMC9901072; doi:10.1186/s13000-023-01300-4)
Supplement: Supplementary file 1 — Additional file 1: Table S1. Overview of the sample size and results of survival analyses for each tumor type. Table S2. Correlations between the studied markers and the selected clinico-pathological parameters for different tumor types. [file 13000_2023_1300_MOESM1_ESM.docx]

Supplementary files:

Table S1: Overview of the sample size and results of survival analyses for each tumor type.

|  |  | **IMP3** | | | **IMP2**  **clone OTI3F9** | | | **IMP2**  **clone EPR6741(B)** | | | | | |
| --- | --- | --- | --- | --- | --- | --- | --- | --- | --- | --- | --- | --- | --- |
| Diagnosis | Survival (cens/complete) | N  negative | N  positive | *p*-value | N  negative | N  positive | *p*-value | N  negative | N  positive | | | *p*-value | |
| HGSC (FU = 110) | |  |  |  |  |  |  |  |  | | |  | |
|  | OS | 28/9 | 58/15 | 0.429 | 1/1 | 85/23 | *NULL* | 0/0 | 86/24 | | | *NULL* | |
|  | RFS | 9/28 | 17/56 | 0.135 | 0/2 | 26/82 | *NULL* | 0/0 | 26/84 | | | *NULL* | |
|  | LFS | 11/26 | 42/31 | 0.106 | 1/1 | 52/56 | *NULL* | 0/0 | 53/57 | | | *NULL* | |
|  | MFS | 24/13 | 47/26 | 0.568 | 1/1 | 70/38 | *NULL* | 0/0 | 71/39 | | | *NULL* | |
| LGSC (FU = 80) | |  |  |  |  |  |  |  |  | | |  | |
|  | OS | 44/16 | 16/4 | 0.858 | 1/1 | 59/19 | *NULL* | 2/0 | 58/20 | | | *NULL* | |
|  | RFS | 36/24 | 8/12 | **0.033** | 1/1 | 43/35 | *NULL* | 2/0 | 42/36 | | | *NULL* | |
|  | LFS | 42/18 | 13/7 | 0.128 | 1/1 | 54/24 | *NULL* | 2/0 | 53/25 | | | *NULL* | |
|  | MFS | 48/12 | 16/4 | 0.557 | 1/1 | 63/15 | *NULL* | 2/0 | 62/16 | | | *NULL* | |
| mSBT (FU = 27) | |  |  |  |  |  |  |  |  | | |  | |
|  | OS | 19/2 | 4/2 | 0.076 | 0/0 | 23/4 | *NULL* | 0/0 | 23/4 | | | *NULL* | |
|  | RFS | 15/6 | 3/3 | 0.939 | 0/0 | 18/9 | *NULL* | 0/0 | 18/9 | | | *NULL* | |
|  | LFS | 16/5 | 5/1 | *NULL* | 0/0 | 21/6 | *NULL* | 0/0 | 21/6 | | | *NULL* | |
|  | MFS | 20/1 | 5/1 | *NULL* | 0/0 | 25/2 | *NULL* | 0/0 | 25/2 | | | *NULL* | |
| EC (FU = 50) | |  |  |  |  |  |  |  |  | | |  | |
|  | OS | 30/0 | 19/1 | *NULL* | 5/0 | 44/1 | *NULL* | 13/0 | 36/1 | | | *NULL* | |
|  | RFS | 28/2 | 19/1 | *NULL* | 5/0 | 42/3 | *NULL* | 12/1 | 35/2 | | | *NULL* | |
|  | LFS | 29/1 | 19/1 | *NULL* | 5/0 | 43/2 | *NULL* | 13/0 | 35/2 | | | *NULL* | |
|  | MFS | 30/0 | 19/1 | *NULL* | 5/0 | 44/1 | *NULL* | 13/0 | 36/1 | | | *NULL* | |
| CCC (FU = 106) | |  |  |  |  |  |  |  |  | | |  | |
|  | OS | 27/6 | 53/20 | 0.168 | 8/4 | 72/22 | 0.342 | 5/3 | 75/23 | | | 0.468 | |
|  | RFS | 21/12 | 50/23 | 0.526 | 10/2 | 61/33 | 0.083 | 6/2 | 65/33 | | | 0.183 | |
|  | LFS | 28/5 | 56/17 | 0.346 | 12/0 | 72/22 | 0.116 | 8/0 | 76/22 | | | 0.147 | |
|  | MFS | 27/6 | 65/8 | 0.606 | 10/2 | 82/12 | 0.739 | 5/3 | 87/11 | | | 0.420 | |
| MBT (FU = 63)* | |  |  |  |  |  |  |  |  | | |  | |
|  | OS | 11/0 | 50/2 | *NULL* | 1/1 | 58/1 | *NULL* | 1/1 | 58/1 | | | *NULL* | |
|  | RFS | 11/0 | 52/0 | *NULL* | 2/0 | 59/0 | *NULL* | 2/0 | 59/0 | | | *NULL* | |
|  | LFS | 11/0 | 52/0 | *NULL* | 2/0 | 59/0 | *NULL* | 2/0 | 59/0 | | | *NULL* | |
|  | MFS | 11/0 | 52/0 | *NULL* | 2/0 | 59/0 | *NULL* | 2/0 | 59/0 | | | *NULL* | |
| MC (FU = 27)* | |  |  |  |  |  |  |  |  | | |  | |
|  | OS | 3/1 | 21/2 | *NULL* | 1/0 | 21/3 | *NULL* | 2/0 | 20/3 | | | *NULL* | |
|  | RFS | 2/2 | 16/7 | *NULL* | 1/0 | 16/8 | *NULL* | 1/1 | 16/7 | | | *NULL* | |
|  | LFS | 4/0 | 17/6 | *NULL* | 1/0 | 19/5 | *NULL* | 1/1 | 19/4 | | | *NULL* | |
|  | MFS | 4/0 | 21/2 | *NULL* | 1/0 | 22/2 | *NULL* | 2/0 | 21/2 | | | *NULL* | |
| HGSC = high grade serous carcinoma, LGSC = low grade serous carcinoma, mSBT = micropapillary serous borderline tumor, EC = endometrioid carcinoma, CCC = clear-cell carcinoma, MBT = mucinous borderline tumor, MC = mucinous carcinoma. Cens = censored (event of interest was not observed or information is incomplete), complete = patient had an event of interest, FU = known follow-up, OS = overall survival, RFS = relapse-free survival, LFS = local recurrence=free survival, MFS = metastasis-free survival, *some cases in subgroup of MBT (2) and MC (2) had missing value of expression of IMP2 clones, NULL = lack of value, *p*-values are based on log rank test, significant values are indicated bold. | | | | | | | | | | | | | |
|  | | | | | | | | | |  |  | |  |

Table S2: Correlations between the studied markers and the selected clinico-pathological parameters for different tumor types.

| IMP3 |  |  |  |  |  |  |  |  |  |  |  |  |  |  |  |  |  |  |  |  |  |
| --- | --- | --- | --- | --- | --- | --- | --- | --- | --- | --- | --- | --- | --- | --- | --- | --- | --- | --- | --- | --- | --- |
| **Diagnoses** | **HGSC (N=114)** | | | **LGSC (N=100)** | | | **mSBT (N=41)** | | | **EC ( N=4)** | | | **CCC (N=124)** | | | **MBT (N=75)** | | | **MC (N=34)** | | |
| **Characteristics** | **Negative (N = 34)** | **Positive (N = 80)** | ***p*-value** | **Negative (N = 74)** | **Positive (N = 26)** | ***p*-value** | **Negative (N = 31)** | **Positive (N = 10)** | ***p*-value** | **Negative (N = 27)** | **Positive (N = 27)** | ***p*-value** | **Negative (N = 38)** | **Positive (N = 86)** | ***p*-value** | **negative (N = 14)** | **positive (N = 61)** | ***p*-value** | **negative (N = 6)** | **positive (N = 28)** | ***p*-value** |
| **Age (median = 58)** |  |  | 0.215a |  |  | 0.352a |  |  | 1.000b |  |  | 0.387a |  |  | 0.378a |  |  | 0.723a |  |  | 0.672b |
| ˂ median | 17 | 30 |  | 42 | 13 |  | 14 | 6 |  | 15 | 18 |  | 10 | 31 |  | 8 | 32 |  | 2 | 12 |  |
| ≥ median | 17 | 50 |  | 25 | 12 |  | 9 | 3 |  | 11 | 8 |  | 26 | 55 |  | 5 | 25 |  | 4 | 14 |  |
| *NA* | *0* | *0* |  | *7* | *1* |  | *8* | *1* |  | *1* | *1* |  | *2* | *0* |  | *1* | *4* |  | *0* | *2* |  |
| **FIGO** |  |  | 0.383b |  |  | 0.119a |  |  | 0.691 |  |  | 0.609b |  |  | 0.373a |  |  | NULL |  |  | 1.000b |
| Low (I+II) | 3 | 13 |  | 12 | 9 |  | 8 | 4 |  | 25 | 23 |  | 30 | 60 |  | 14 | 59 |  | 5 | 22 |  |
| High (III+IV) | 31 | 65 |  | 40 | 13 |  | 12 | 4 |  | 1 | 3 |  | 6 | 19 |  | 0 | 1 |  | 1 | 6 |  |
| *NA* | *0* | *2* |  | *22* | *4* |  | *11* | *2* |  | *1* | *1* |  | *2* | *7* |  | *0* | *1* |  | *0* | *0* |  |
| **T stage** |  |  | 0.054a |  |  | 0.132a |  |  | 0.691 |  |  | 1.000b |  |  | 0.349a |  |  | NULL |  |  | 1.000b |
| Low (T1+T2) | 4 | 22 |  | 12 | 9 |  | 8 | 4 |  | 25 | 24 |  | 31 | 63 |  | 14 | 59 |  | 5 | 22 |  |
| High (T3+T4) | 30 | 55 |  | 39 | 13 |  | 12 | 4 |  | 1 | 2 |  | 5 | 17 |  | 0 | 1 |  | 1 | 6 |  |
| *Tx/NA* | *0* | *3* |  | *23* | *4* |  | *11* | *2* |  | *1* | *1* |  | *2* | *6* |  | *0* | *1* |  | *0* | *0* |  |
| **N stage** |  |  | 0.478a |  |  | 1.000b |  |  | 1.000b |  |  | NULL |  |  | 0.668a |  |  | NULL |  |  | 1.000b |
| N0 | 7 | 22 |  | 14 | 4 |  | 8 | 4 |  | 22 | 18 |  | 14 | 39 |  | 1 | 8 |  | 3 | 9 |  |
| N1 | 14 | 30 |  | 16 | 4 |  | 2 | 1 |  | 0 | 1 |  | 1 | 6 |  | 0 | 0 |  | 0 | 3 |  |
| *Nx/NA* | *13* | *28* |  | *44* | *18* |  | *21* | *5* |  | *5* | *8* |  | *23* | *41* |  | *13* | *53* |  | *3* | *16* |  |
| **M stage** |  |  | 0.593a |  |  | 1.000b |  |  | NULL |  |  | NULL |  |  | 1.000b |  |  | NULL |  |  | NULL |
| M0 | 21 | 48 |  | 48 | 22 |  | 18 | 10 |  | 26 | 21 |  | 2 | 4 |  | 13 | 57 |  | 5 | 28 |  |
| M1 | 7 | 21 |  | 2 | 0 |  | 0 | 0 |  | 0 | 1 |  | 0 | 1 |  | 0 | 1 |  | 1 | 0 |  |
| *Mx/NA* | *6* | *11* |  | *24* | *4* |  | *13* | *2* |  | *1* | *5* |  | *36* | *81* |  | *1* | *3* |  | *0* | *0* |  |
| IMP2 clone OTI3F9 |  |  |  |  |  |  |  |  |  |  |  |  |  |  |  |  |  |  |  |  |  |
| **Diagnoses** | **HGSC (N=114)** | | | **LGSC (N=100)** | | | **mSBT (N=41)** | | | **EC ( N=54)** | | | **CCC (N=124)** | | | **MBT (N=73)** | | | **MC (N=32)** | | |
| **Characteristics** | **Negative (N = 0)** | **Positive (N = 114)** | ***p*-value** | **Negative (N = 1)** | **Positive (N = 99)** | ***p*-value** | **Negative (N = 0)** | **Positive (N = 41)** | ***p*-value** | **Negative (N = 3)** | **Positive (N = 51)** | ***p*-value** | **Negative (N = 14)** | **Positive (N = 110)** | ***p*-value** | **negative (N = 3)** | **positive (N = 70)** | ***p*-value** | **negative (N = 1)** | **positive (N = 31)** | ***p*-value** |
| **Age (median = 58)** |  |  | NULL |  |  | NULL |  |  | NULL |  |  | 0.546b |  |  | 0.540b |  |  | 0.127b |  |  | NULL |
| ˂ median | 0 | 47 |  | 0 | 54 |  | 0 | 20 |  | 1 | 32 |  | 3 | 38 |  | 0 | 39 |  | 0 | 14 |  |
| ≥ median | 0 | 57 |  | 1 | 37 |  | 0 | 12 |  | 2 | 17 |  | 10 | 71 |  | 2 | 27 |  | 1 | 16 |  |
| *NA* | *0* | *0* |  | *0* | *8* |  | *0* | *9* |  | *0* | *2* |  | *1* | *1* |  | *1* | *4* |  | *0* | *1* |  |
| **FIGO** |  |  | NULL |  |  | NULL |  |  | NULL |  |  | 1.000b |  |  | 1.000b |  |  | NULL |  |  | NULL |
| Low (I+II) | 0 | 16 |  | 0 | 21 |  | 0 | 12 |  | 3 | 45 |  | 10 | 80 |  | 3 | 68 |  | 1 | 26 |  |
| High (III+IV) | 0 | 96 |  | 1 | 52 |  | 0 | 16 |  | 0 | 4 |  | 3 | 22 |  | 0 | 1 |  | 0 | 5 |  |
| *NA* | *0* | *2* |  | *0* | *26* |  | *0* | *13* |  | *0* | *2* |  | *1* | *8* |  | *0* | *1* |  | *0* | *0* |  |
| **T stage** |  |  | NULL |  |  | NULL |  |  | NULL |  |  | 1.000b |  |  | 1.000b |  |  | NULL |  |  | NULL |
| Low (T1+T2) | 0 | 26 |  | 0 | 21 |  | 0 | 12 |  | 3 | 46 |  | 10 | 84 |  | 3 | 68 |  | 1 | 26 |  |
| High (T3+T4) | 0 | 85 |  | 1 | 51 |  | 0 | 16 |  | 0 | 3 |  | 3 | 19 |  | 0 | 1 |  | 0 | 5 |  |
| *Tx/NA* | *0* | *3* |  | *0* | *27* |  | *0* | *13* |  | *0* | *2* |  | *1* | *7* |  | *0* | *1* |  | *0* | *0* |  |
| **N stage** |  |  | NULL |  |  | NULL |  |  | NULL |  |  | NULL |  |  | 0.514b |  |  | NULL |  |  | NULL |
| N0 | 0 | 29 |  | 1 | 17 |  | 0 | 12 |  | 2 | 38 |  | 5 | 48 |  | 1 | 7 |  | 1 | 11 |  |
| N1 | 0 | 44 |  | 0 | 20 |  | 0 | 3 |  | 0 | 1 |  | 1 | 6 |  | 0 | 0 |  | 0 | 3 |  |
| *Nx/NA* | *0* | *41* |  | *0* | *62* |  | *0* | *26* |  | *1* | *12* |  | *8* | *56* |  | *2* | *61* |  | *0* | *17* |  |
| **M stage** |  |  | NULL |  |  | NULL |  |  | NULL |  |  | NULL |  |  | NULL |  |  | NULL |  |  | NULL |
| M0 | 0 | 69 |  | 0 | 70 |  | 0 | 26 |  | 3 | 44 |  | 0 | 6 |  | 3 | 65 |  | 1 | 31 |  |
| M1 | 0 | 28 |  | 1 | 1 |  | 0 | 0 |  | 0 | 1 |  | 0 | 1 |  | 0 | 1 |  | 0 | 0 |  |
| *Mx/NA* | *0* | *17* |  | *0* | *28* |  | *0* | *15* |  | *0* | *6* |  | *13* | *103* |  | *0* | *4* |  | *0* | *0* |  |
| IMP2 clone EPR6741(B) | |  |  |  |  |  |  |  |  |  |  |  |  |  |  |  |  |  |  |  |  |
| **Diagnoses** | **HGSC (N=114)** | | | **LGSC (N=100)** | | | **mSBT (N=41)** | | | **EC ( N=54)** | | | **CCC (N=124)** | | | **MBT (N=73)** | | | **MC (N=32)** | | |
| **Characteristics** | **Negative (N = 0)** | **Positive (N = 114)** | ***p*-value** | **Negative (N = 2)** | **Positive (N = 98)** | ***p*-value** | **Negative (N = 0)** | **Positive (N = 41)** | ***p*-value** | **Negative (N = 13)** | **Positive (N = 41)** | ***p*-value** | **Negative (N = 9)** | **positive (N = 115)** | ***p*-value** | **negative (N = 3)** | **positive (N = 70)** | ***p*-value** | **negative (N = 3)** | **positive (N = 29)** | ***p*-value** |
| **Age (median = 58)** |  |  | NULL |  |  | NULL |  |  | NULL |  |  | 0.406a |  |  | 0.265b |  |  | 1.000b |  |  | 1.000b |
| ˂ median | 0 | 47 |  | 1 | 54 |  | 0 | 20 |  | 7 | 26 |  | 1 | 40 |  | 1 | 38 |  | 1 | 13 |  |
| ≥ median | 0 | 57 |  | 1 | 36 |  | 0 | 12 |  | 6 | 13 |  | 7 | 74 |  | 1 | 28 |  | 2 | 15 |  |
| *NA* | *0* | *0* |  | *0* | *8* |  | *0* | *9* |  | *0* | *2* |  | *1* | *1* |  | *1* | *4* |  | *0* | *1* |  |
| **FIGO** |  |  | NULL |  |  | NULL |  |  | NULL |  |  | 0.561b |  |  | 1.000b |  |  | NULL |  |  | 1.000b |
| Low (I+II) | 0 | 16 |  | 2 | 19 |  | 0 | 12 |  | 13 | 35 |  | 6 | 84 |  | 3 | 68 |  | 3 | 24 |  |
| High (III+IV) | 0 | 96 |  | 0 | 53 |  | 0 | 16 |  | 0 | 4 |  | 2 | 23 |  | 0 | 1 |  | 0 | 5 |  |
| *NA* | *0* | *2* |  | *0* | *26* |  | *0* | *13* |  | *0* | *2* |  | *1* | *8* |  | *0* | *1* |  | *0* | *0* |  |
| **T stage** |  |  | NULL |  |  | NULL |  |  | NULL |  |  | 0.564b |  |  | 0.657b |  |  | NULL |  |  | 1.000b |
| Low (T1+T2) | 0 | 26 |  | 2 | 19 |  | 0 | 12 |  | 13 | 36 |  | 7 | 87 |  | 3 | 68 |  | 3 | 24 |  |
| High (T3+T4) | 0 | 85 |  | 0 | 52 |  | 0 | 16 |  | 0 | 3 |  | 1 | 21 |  | 0 | 1 |  | 0 | 5 |  |
| *Tx/NA* | *0* | *3* |  | *0* | *27* |  | *0* | *13* |  | *0* | *2* |  | *1* | *7* |  | *0* | *1* |  | *0* | *0* |  |
| **N stage** |  |  | NULL |  |  | NULL |  |  | NULL |  |  | 1.000b |  |  | 0.315b |  |  | NULL |  |  | 1.000b |
| N0 | 0 | 29 |  | 2 | 16 |  | 0 | 12 |  | 10 | 30 |  | 2 | 51 |  | 1 | 7 |  | 3 | 9 |  |
| N1 | 0 | 44 |  | 0 | 20 |  | 0 | 3 |  | 0 | 1 |  | 1 | 6 |  | 0 | 0 |  | 0 | 3 |  |
| *Nx/NA* | *0* | *41* |  | *0* | *62* |  | *0* | *26* |  | *3* | *10* |  | *6* | *58* |  | *2* | *63* |  | *0* | *17* |  |
| **M stage** |  |  | NULL |  |  | NULL |  |  | NULL |  |  | 1.000b |  |  | NULL |  |  | NULL |  |  | NULL |
| M0 | 0 | 69 |  | 2 | 68 |  | 0 | 26 |  | 13 | 34 |  | 0 | 6 |  | 3 | 65 |  | 3 | 29 |  |
| M1 | 0 | 28 |  | 0 | 2 |  | 0 | 0 |  | 0 | 1 |  | 0 | 1 |  | 0 | 1 |  | 0 | 0 |  |
| *Mx/NA* | *0* | *17* |  | *0* | *28* |  | *0* | *15* |  | *0* | *6* |  | *9* | *108* |  | *0* | *4* |  | *0* | *0* |  |

*P-values are based on the Pearson chi-squared test (^a^) or the Fisher exact test (^b^). Only known values are included into the analyses (NA = not available, NULL = absence of value).* HGSC = high grade serous carcinoma, LGSC = low grade serous carcinoma, mSBT = micropapillary serous borderline tumor, EC = endometrioid carcinoma, CCC = clear-cell carcinoma, MBT = mucinous borderline tumor, MC = mucinous carcinoma.
